# Chronic Antagonism of the Mineralocorticoid Receptor Ameliorates Hypertension and End Organ Damage in a Rodent Model of Salt-Sensitive Hypertension

**DOI:** 10.3109/10641963.2011.566956

**Published:** 2011-09-27

**Authors:** Xiaoyan Zhou, Martin F Crook, Wanda Sharif-Rodriguez, Yonghua Zhu, Zadok Ruben, Yi Pan, Olga Urosevic-Price, Li Wang, Amy M Flattery, Gail Forrest, Daphne Szeto, Huawei Zhao, Sophie Roy, Michael J Forrest

**Affiliations:** 1 Department of Cardiovascular Diseases, Merck Research Laboratories, Merck and Company, Rahzvay, New Jersey, USA; 2 Department of Central Pharmacology, Merck Research Laboratories, Merck and Company, Rahzvay, NJ, USA; 3 Patoximed Consultants, Westfield, New Jersey, USA

**Keywords:** mineralocorticoid receptor antagonist, hypertension, end organ protection, eplerenone, Dahl salt-sensitive rats

## Abstract

We investigated the effects of chronic mineralocorticoid receptor blockade with eplerenone on the development and progression of hypertension and end organ damage in Dahl salt-sensitive rats. Eplerenone significantly attenuated the progressive rise in systolic blood pressure (SBP) (204 ± 3 vs. 179±3 mmHg, p < 0.05), reduced proteinuria (605.5 ± 29.6 vs. 479.7 ± 26.1 mg/24h, p < 0.05), improved injury scores of glomeruli, tubules, renal interstitium, and vasculature in Dahl salt-sensitive rats fed a high-salt diet. These results demonstrate that mineralocorticoid receptor antagonism provides target organ protection and attenuates the development of elevated blood pressure (BP) in a model of salt-sensitive hypertension.

## INTRODUCTION

Aldosterone plays a critical role in regulating body fluid, electrolytes, and blood pressure (BP) homeostasis, which is achieved by promoting the reabsorption of sodium and water and the secretion of potassium in the connecting tubules and collecting ducts in the kidneys. Accumulated evidence suggests that mineralocorticoid receptor (MR) activation mediated by increased aldosterone plays a crucial role in the development and progression of hypertension and end organ damage (1-3). Extensive animal and clinical studies have demonstrated that MR antagonism lowers BP (4-11) and provides cardiac, vascular, and renal protection via both hemodynamic and non-hemodynamic mechanisms (5-8,11-16). Moreover, the Eplerenone Post-Acute Myocardial Infarction Heart Failure Efficacy and Survival Study (EPHESUS) demonstrated the addition of eplerenone, a selective MR antagonist, to standard medical therapy reduced morbidity and mortality among patients with acute myocardial infarction complicated by left ventricular dysfunction and heart failure (17).

Hypertension affects approximately 50 million individuals in the United States (18) and approximately 1 billion people worldwide (19). A substantial proportion of individuals with hypertension in industrialized countries consume high levels of salt and Weinberger reported that approximately 50% of individuals with essential hypertension are salt-sensitive (20). Abnormalities in aldosterone-mediated MR activation are associated with salt-sensitivity and hypertension (21-25). The Dahl salt-sensitive (Dahl SS) rat, a rodent model of salt-sensitive hypertension, has been widely used to investigate the molecular mechanisms underlying the development of salt-induced hypertension and to evaluate the efficacy of pharmacologic interventions in salt-sensitive hypertension (26-30). The selective MR antagonist, eplerenone, has been evaluated extensively in Dahl SS rats on a high-salt diet. However, reports on its efficacy for lowering BP were diverse: some studies reported a marked attenuation of the increase in BP (4,7,8), whereas other studies reported no effects on BP in response to salt-loading in Dahl SS rats (14,15).

Importantly, none of these studies have comprehensively investigated the effects of eplerenone on indices of end organ damage. In addition, few studies have assessed the BP lowering efficacy of eplerenone in Dahl SS rats on low-salt diet. To this end, the present study was designed to evaluate the effects of chronic MR antagonism with eplerenone on the development and progression of hypertension using radiotelemetry and to assess the effects of eplerenone on end organ structure and function and renal injury biomarkers in Dahl SS rats maintained on high- or low-salt diets.

## MATERIALS AND METHODS

Dahl SS rats (Harlan Labs. Inc: Indianapolis, IN, USA) were housed in a temperature- and humidity-controlled room with a 12:12-h dark-light cycle with food and water provided *ad libitum*. Rodent diets (#7034, low-salt diet, containing 0.3% NaCl; TD.92034, high-salt diet, containing 4% NaCl) were purchased from Harlan Teklad (Madison, WI, USA). Eplerenone, purified from Inspra tablets (Pfizer Inc., New York, NY, USA) was used to prepare eplerenone-medicated diets (based on Harlan Tekald #7034 and TD.92034) by Research Diets, Inc. (New Brunswick, NJ, USA). The eplerenone concentration in the diet was calculated to give a daily dose of 100 mg·kg^−1^d^−1^ eplerenone based on food intake relative to body weight.

All procedures utilizing experimental animals were conducted in accordance with the Guide for the Care and Use of Laboratory Animals, and experimental protocols were approved in advance by the Institutional Animal Care and Use Committee at Merck Research Laboratories, Rahway, NJ.

### Radiotelemetry Implant Surgery

Male Dahl SS rats (15 weeks old) were anesthetized with isoflurane and premedicated with buprenorphine (0.03 mg·kg^−1^, s.c.: Reckitt Benckiser healthcare Ltd., Hull, UK) prior to surgery. Telemetry devices (TA11PA-C40, Data Sciences International, DSI, St. Paul, MN, USA) were aseptically placed in a subcutaneous pocket on one side of the body with the catheter inserted into the descending aorta via the femoral artery. Penicillin G (150,000 U·kg^−1^, s.c. Bimeda Inc., Irwindale, CA, USA) was administered at the end of surgery. Rats were allowed to recover for 2 to 3 weeks prior to experimentation.

### Experimental Design

Dahl SS rats with implanted radiotelemetry devices were housed individually in Nalgene metabolism cages (Braintree Scientific, Inc., Braintree, MA, USA) following a 1-week acclimation period. All animals were maintained on Harlan Teklad diet #7034 (0.3% NaCl) prior to study. The rats were randomly divided into four groups: Group 1, low-salt diet (0.3% NaCl), n = 8; Group 2, low-salt diet plus eplerenone 100 mg·kg^−1^d^−1^, n = 8; Group 3, high-salt diet (4% NaCl), n = 8; Group 4, high-salt diet plus eplerenone 100 mg·kg^−1^d^−1^, n = 8. Animals were maintained in these treatment groups for an 8-week period.

### BP Measurement, Renal Function Assessment, and Kidney Injury Biomarkers Detection

Radiotelemetry signals were collected and analyzed using a DSI Dataquest System Version 4.1 (Data Sciences International, DSI, St. Paul, MN, USA). Mean, systolic, diastolic, and pulse arterial BPs and heart rate were determined on a beat-by-beat basis. Data was collected for 30 min every hour and was reported as average values for each animal over a 24-h period. Twenty-four hour food and water intake and urine output were monitored once a week during the study. Urinary electrolytes (including Na, K, and Cl), creatinine, and protein concentration were assessed by a Roche Modular Chemistry System (Roche Diagnostics, Indianapolis, IN, USA). Kidney injury biomarkers including lipocalin-2 (LPN), osteopontin (OPN), kidney injury molecule-1 (KIM-1), and renal papillary antigen 1 (RPA-1) were detected using a Kidney Injury Panel 1(rat) kit and Argutus AKI (rat) kit from Meso Scale Discovery, LLC (Gaithersburg, MA, USA). All these biomarkers have been validated in animal studies (31).

### Serum Aldosterone, Plasma Electrolytes, Creatinine, and Eplerenone Concentration Measurement

Upon study termination, blood was collected by jugular venepuncture from conscious animals and was used for measuring serum aldosterone by ELISA (IBL, Hamburg, Germany). Thereafter, animals were euthanized by CO2 inhalation and cardiac puncture was performed to collect blood for determination of plasma electrolytes and creatinine (Roche Diagnostics, Indianapolis, IN, USA) and eplerenone levels (determined by LC/MS/MS following protein precipitation with acetonitrile).

### Histopathology

Kidney and heart tissues were harvested from all animals, fixed in Prefer (Anatech LTD., Battle Creek, MI, USA) for at least 24 h, and were paraffin-embedded. Tissue sections were stained with hematoxylin and eosin (H&E) and the heart also with Masson's trichrome strain (for collagen). The severity of histopathologic changes in renal tubules, interstitium, vasculature, and glomeruli were graded on a 1 to 5 scale corresponding to minimal, mild, moderate, marked, and severe (32). Sections from both kidneys were examined and the final scores composite are a composite from all sections of each slice. Separate deparaffmized heart tissue sections were stained with Masson trichrome, which stains collagen-enriched areas as blue and cellular elements as red. Collagen deposition in the ventricle wall and perivascular area was graded on a 1 to 5 scale corresponding to minimal, mild, moderate, marked, and severe, based on the blue stained area size and intensity.

### Statistical Analyses

All data are presented as means ± standard error of the mean (SE). A repeated measure analysis of variance (rmANOVA) was used for time course data analysis. One-way ANOVA followed by Newman-Keuls post-hoc test was used for comparisons of only one time point values in all groups. P value of <0.05 was considered to be of statistical significance.

## RESULTS

### Effects of Eplerenone on Blood Pressure and Heart Rate

**Figure 1:**
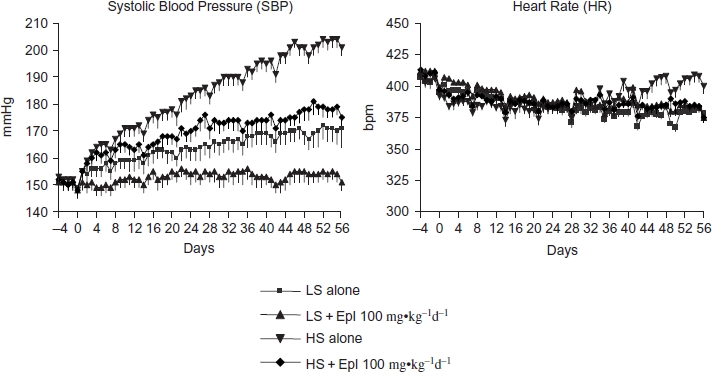
Effects of chronic eplerenone treatment on BP and heart rate in Dahl SS rats. Data are mean ± SEM (*n* = 8 for each group). Abbreviations: LS - low salt; HS - high salt; Epl - eplerenone. High salt statistically significantly increased sBP after 1 week of salt-loading (p < 0.05, HS alone vs. LS alone). sBP in LS + Epl 100 mg⋅kg^−1^d^−1^ and HS + Epl 100 mg-kg^−1^d^−1^ group was statistically significantly lower than the LS alone (since the first week of treatment) and HS alone group (since the second week of treatment), respectively (p < 0.05). Heart rate was statistically significantly higher in the HS alone group than the other three groups in the last 3 weeks of the study (p < 0.05) (color figure available online).

The baseline SBP of Dahl SS rats, while maintained on a low (0.3% NaCl) dietary salt and measured 2 to 3 weeks post-implantation of telemetry devices, was 151 ± 3 mmHg (Figure 1). Systolic BP gradually increased by 18 ± 3 mmHg over the subsequent 8 weeks (from age 18 weeks to 25 weeks). This increase reflects the spontaneous development of hypertension characteristic of this rat strain. As expected, consumption of a high-salt (4% NaCl) diet accelerated the progression of hypertension. Thus, SBP increased by 52 ± 2 mmHg from 152 ± 3 mmHg to 204 ± 3 mmHg over an 8-week period of high-salt feeding. The extent of BP elevation in response to a high-salt diet was similar when measured during the day and night periods (data not shown). Chronic treatment with eplerenone (100 mg·kg^−1^d^−1^) completely blocked the SBP increase in animals fed a low-salt diet (170 ± 5 mmHg vs. 154 ± 3 mmHg, p < 0.05) and significantly blunted the progression of hypertension in animals fed a high-salt diet (204 ± 3 mmHg vs. 179 ± 3 mmHg, p < 0.05) (Figure 1). Heart rates declined by less than 10% during the course of study and for the most part were not significantly different among groups receiving low or high salt diet with or without eplerenone (Figure 1). There was a small increase in heart rate during the last 3 weeks for those animals on the high-salt diet that was not apparent in those animals receiving high salt plus eplerenone. Interestingly, we observed a consistent transient decrease in BP and heart rate contemporaneous with the weekly collection of urine samples from these animals. The magnitude of the decrease was similar for all groups of animals and we attribute these changes to the increased investigator presence during this procedure.

### Renal Excretory Effects of Eplerenone

**Figure 2:**
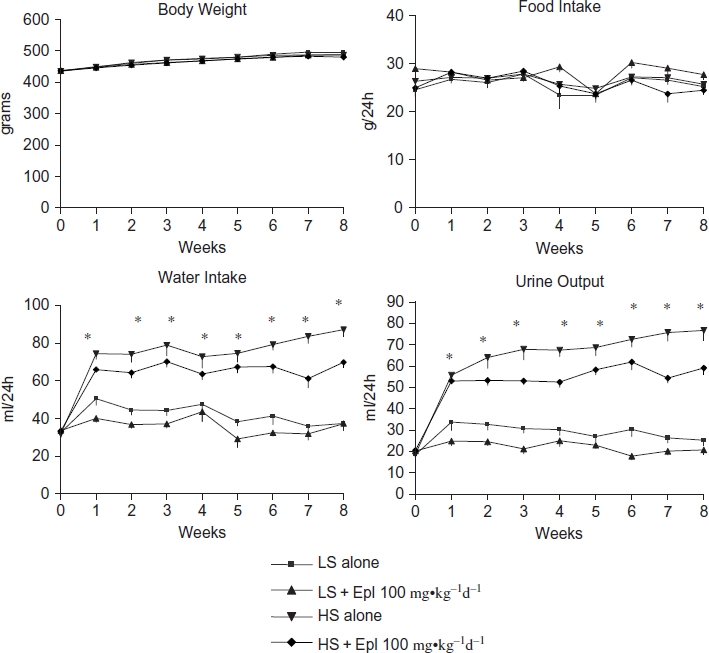
Effects of eplerenone on body weight, food intake, water intake, and urine output in Dahl SS rats. Data are mean ± SEM *﹛n* = 8 for each group). Abbreviations: LS - low salt; HS - high salt; Epl - eplerenone. Body weight and food intake were not statistically significantly different among all groups. Water intake and urine output were statistically significantly greater in the two high-salt diet groups than that in the two low-salt diet groups (*p < 0.05, HS groups vs. LS groups) (color figure available online).

**Figure 3:**
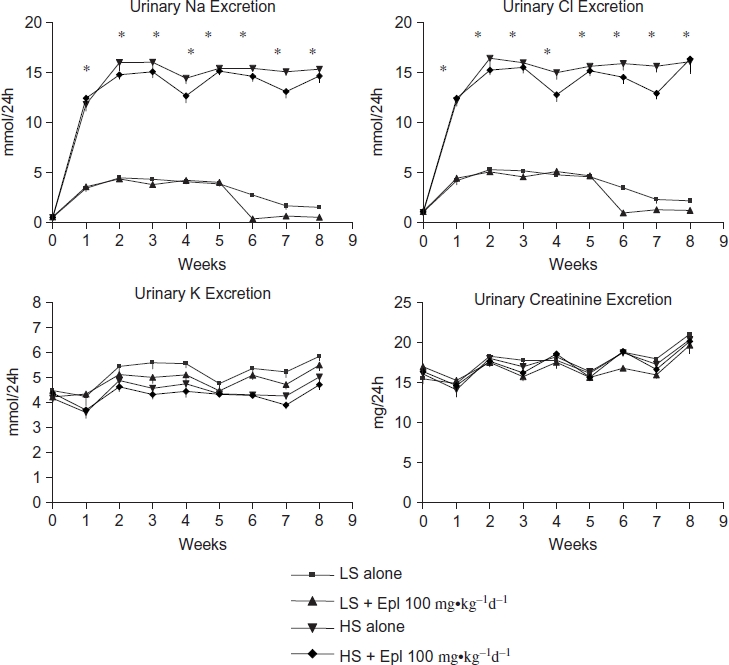
Effects of Eplerenone on urinary electrolytes and creatinine excretion in Dahl SS rats. Data are mean ± SEM (*n* = 8 for each group). Abbreviations: LS - low salt; HS - high salt; Epl - eplerenone. Urinary Na and Cl excretion were statistically significantly greater in the two high-salt diet groups than that in the two low-salt diet groups (*p < 0.05, HS groups vs. LS groups). Urinary K and creatinine excretion were not statistically significantly different among all groups (color figure available online).

**Figure 4:**
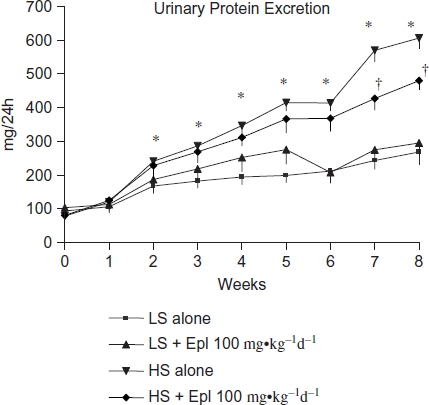
Time course of 24-h urinary protein excretion. Data are mean ± SEM (*n* = 8 for each group). Abbreviations: LS - low salt; HS - high salt; Epl - eplerenone. Urinary protein excretion was statistically significantly greater in the HS alone group than that in the LS alone group (*p < 0.05, HS alone vs. LS alone). Proteinuria was statistically significantly reduced in the HS + Epl 100 mg-kg^−1^d^−1^ group compared with the HS alone group in the 7th and 8th week of the study (†p < 0.05, HS + Epl 100 mg-kg^−1^d^−1^ vs. HS alone) (color figure available online).

The effects of eplerenone on body weight, food and water intake, urine output, and urinary excretion of Na, Cl, K, and creatinine are shown in Figures 2 and 3. Baseline data (reported as week 0) from all groups maintained with low-salt diet was collected 1 week prior to administration of eplerenone and to placing animals on a high- salt diet. Body weight or food intake did not vary among the groups. Water intake, urine output, and urinary excretion of Na and Cl were statistically significantly greater in the two high-salt diet groups than in the low-salt diet groups. Eplerenone did not exhibit natriuretic effects on Dahl SS rats on either a low-salt or high-salt diet at steady state. Urinary excretion of K and creatinine were not statistically significantly different between the groups. In addition, we tested the acute natriuretic effect of eplerenone in a separate cohort of age-matched Dahl SS rats and found that eplerenone elicited a significant natriuretic effect in the low-salt diet condition (24 h urinary Na excretion of 1.92 ± 0.37 mmol/24 h in animals that received low salt diet plus eplerenone 100 mg-kg^−1^d^−1^ vs. 1.26 ± 0.34 mmol/24 h in animals that received low salt diet alone, n = 5 per group, p < 0.05). However, under conditions of high-salt intake, sodium excretion was equivalent (P > 0.05) in control animals (9.24 ±1.61 mmol/24 h, n = 5) and those that received eplerenone (9.35 ± 0.59 mmol/24 h, n = 5). Urinary protein excretion was markedly increased in Dahl SS rats on the high-salt diet, which was significantly attenuated (p < 0.05) with eplerenone after treatment for 7 to 8 weeks (Figure 4).

### Biomarkers of Kidney Injury

**Figure 5:**
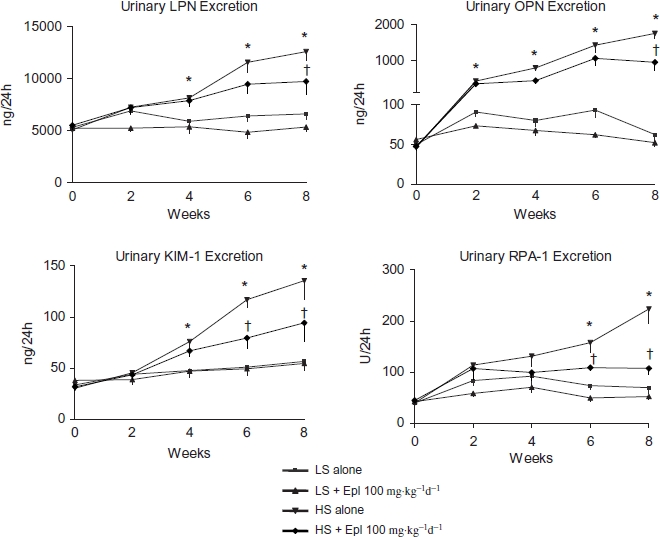
Time course of 24-h urinary kidney injury biomarkers. Data are mean ± SEM (*n* = 8 for each group). Abbreviations: LS - low salt; HS - high salt; Epl - eplerenone; LPN - lipocalin-2; OPN -osteopontin; KIM-1 -kidney biomarker molecule-1; RPA-1 -renal papillary antigen 1. All these biomarkers were increased as early as 2-4 weeks after salt-loading and were statistically significantly diminished by eplerenone treatment towards the end of the study. (*p < 0.05, HS alone vs. LS alone; †p < 0.05, HS + Epl 100 mg-kg^−1^d^−1^ vs. HS alone) (color figure available online).

Urinary LPN and OPN are biomarkers of renal tubular epithelial injury. KIM-1, also known as TIM-1 (T cell immunoglobulin mucin domains—1), is a protein that is predominately expressed in proximal tubules and is a sensitive biomarker for proximal tubular injury. RPA-1 is considered as a collecting duct/renal papilla injury biomarker. All these biomarkers were increased as early as 2-4 weeks after salt-loading and were significantly diminished by eplerenone treatment towards the end of the study (Figure 5).

### Serum Aldosterone, Plasma Electrolytes, Creatinine, and Eplerenone Levels

**Figure 6:**
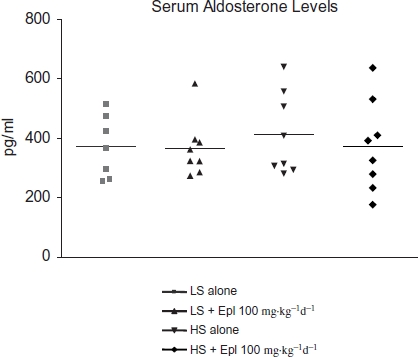
Serum aldosterone level at the end of an 8-week treatment. Data are mean ± SEM (*n* = 8 for each group). Abbreviations: LS - low salt; HS - high salt; Epl - eplerenone. There was no statistically significant difference in serum aldosterone level among all groups (color figure available online).

Serum aldosterone levels and plasma Na, K, and creatinine concentrations were not different among groups (Figure 6 and Table 1). There was a trend towards an increase in plasma K in those animals that received high salt plus eplerenone but this did not reach statistical significance. The plasma eplerenone concentrations in the animals that received low salt and high salt were 77.3 ± 11.6 and 90.6 ± 19.4 nM, respectively (Table 2).

**Table 1:** Plasma creatinine, Na, and K concentration at the end of 8 weeks treatment

	Group 1	Group 2	Group 3	Group 4
	
	LS alone	LS + Epl 100 mg·kg^−1^d^−1^	HS alone	HS + Epl 100 mg·kg^−1^d^−1^
Creatinine (mg/dl)	0.41 ±0.04	0.53±0.07	0.57 ±0.02	0.66±0.11
Na (mmol/1)	141.2±0.83	141.6±0.81	143.3±0.91	142.9±1.40
K (mmol/1)	5.22±0.17	4.93±0.13	5.12±0.18	5.52±0.12

Abbreviations: LS - low salt; HS - high salt; Epl - eplereneone. Data are mean ± SEM (n = 8 in each group).

**Table 2:** Plasma eplerenone level at the end of an 8-week treatment

Group	Eplerenone Level*
low salt + eplerenone 100 mg·kg ^−1^d^−1^	77.3 ± 11.6nM
high salt + eplerenone 100 mg·kg^−1^d^−1^	90.6 ± 19.4 nM

Data are mean ± SEM (n = 8 in each group).

* Plasma eplerenone level at 3—4 h after food was removed.

### Organ Weights and Histopathologic Findings

**Figure 7:**
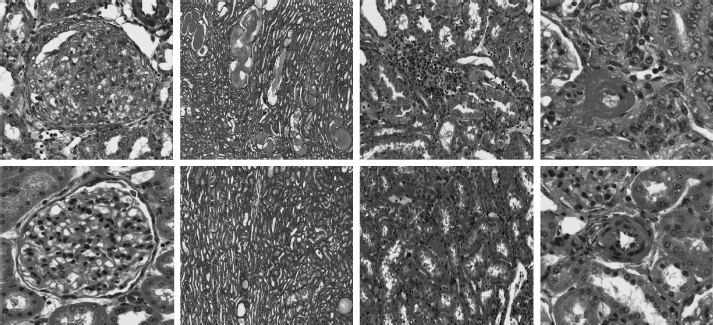
Representative light microscopic findings in glomeruli, renal tubules, interstitium, and vasculature. Upper panel: HS alone, glomerulus, renal tubules, interstitium, and renal artery (left→aright). Lower panel: HS + Epl 100 mg-kg^−1^d^−1^;, glomerulus, renal tubules, interstitium, and renal artery (left→right). High salt alone group shows excess mesangium and decreased capillary lumen in glomerulus, dilated tubules filled with proteinaceous materials, subendothelial fibrinoid material and endothelial proliferation in renal artery, and interstitium infiltration with mononuclear inflammatory cells. HS + Epl 100 mg-kg^−1^d^−1^ group exhibits normal appearance of glomerulus, decreased dilation and proteinaceous content in renal tubules, normal vascular appearance, and no infiltration with mononuclear inflammatory cells in interstitium (color figure available online).

**Figure 8:**
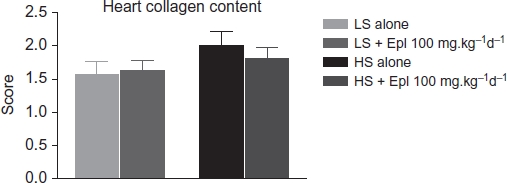
Heart collagen content in all groups. Abbreviations: LS - low salt; HS - high salt; Epl - eplerenone. Heart collagen content was not statistically significantly different among all groups (color figure available online).

Animals on the high-salt diet had significantly increased heart and kidney weights, and eplerenone treatment tended to attenuate these changes (Table 3). Histologic evidence for renal injury was present in rats on either a low- or high-salt diet but was more severe for those animals on the high salt diet (Table 4). Thus, Dahl SS rats on the high-salt diet (Figure 7) exhibited severe focal-segmental or global glomerulosclerosis, perivascular fibrosis, inflammatory cell infiltration, interstitial fibrosis, tubular dilation, and tubular cast formation, and the quantitative injury scores of glomeruli, vasculature, interstitium, and renal tubules were significantly increased. Eplerenone treatment improved the renal histopathologic alterations induced by salt-loading (Table 4). With respect to heart collagen content, since collagen is a normal component of the interstitium, scores 1 and 2 reflect the normal range of histologic variations; salt-loading for 8 weeks did not significantly increase heart collagen content (Figure 8).

**Table 3:** Body and organ weights

	Group 1	Group 2	Group 3	Group 4
	
	LS alone	LS + Epl 100 mg·kg^−1^d^−1^	HS alone	HS + Epl 100 mg·kg^−1^d^−1^
BW,g	495.4±11.9	488.1 ±7.7	486.5±7.1	480.4 ±4.8
HW,g	1.94 ±0.03	1.78±0.04*	2.05 ±0.04*	1.95±0.04
KW,g	3.59±0.12	3.59 ±0.07	4.51 ±0.21*	4.16±0.20
HW/BW, mg/g	3.92±0.09	3.64±0.11	4.22 ±0.09*	4.06±0.10
KW/BW, mg/g	7.24±0.14	7.36 ±0.09	9.26 ±0.35*	8.66 ±0.39

Abbreviations: LS - low salt; HS - high salt; Epl - eplereneone; BW - body weight; HW - heart weight; KW - kidney weight. Data are mean ± SEM (n = 8 in each group).

* p < 0.05 vs. LS alone.

**Table 4:** Renal histopathologic scores

	Group 1	Group 2	Group 3	Group 4

	LS alone	LS + Epl 100 mg·kg^−1^d^−1^	HS alone	HS + Epl 100 mg·kg^−1^d^−1^
Tubules	2.0±0.1	1.5±0.2	2.9 ±0.2*	2.4±0.1†
Interstitium	1.3±0.2	1.3±0.2	2.2±0.1*	1.7±0.1†
Vasculature	0.5±0.1	0.5±0.2	2.3 ±0.3*	0.9±0.3†
Glomeruli	0.6±0.1	0.7 ±0.3	2.2±0.3*	1.0±0.2†

Abbreviations: LS - low salt; HS - high salt; Epl - eplereneone. Data are mean ± SEM (n = 8 in each group).

* p < 0.05 vs. LS alone

† p < 0.05 vs. HS alone.

## DISCUSSION

Our results demonstrate that Dahl SS rats exhibit a slow progression of hypertension when maintained on a low-salt diet, and consumption of a high-salt diet markedly accelerated the development of hypertension. Eplerenone completely blocked and significantly attenuated the progressive rise in SBP in Dahl SS rats under low-salt and high-salt conditions, respectively. This observation concurs with several recent reports that treatment with eplerenone substantially prevented the development of high salt-induced hypertension in Dahl SS rats (4,7,8). However, in contrast to these findings, other investigators reported that eplerenone has no effects on BP in salt-loaded Dahl SS rats (14,15). It is possible that differences in experimental protocol and technique may contribute to these different findings. For example, differences in the age of animals used and in different salt diets employed could influence the time course and magnitude of the hypertension and renal injury. As demonstrated here, the BP lowering efficacy of eplerenone is quite different in animals maintained on low- and high-salt diets. It is well documented that long-term exposure to a high-salt diet from a young age provokes severe hypertension and organ damage (14,15), which may be refractory to mineralocorticoid antagonism. A second possible explanation for the different effects of eplerenone on BP in this and other studies could be related to the techniques used to measure BP. We employed radiotelemetry, generally viewed as the “gold standard” for BP measurement (33), in conscious freely moving animals. However, studies that used the indirect tail cuff plethysmographpy (14,15) carry limitations in accuracy and do not monitor BP during an entire 24-h period such that minor differences in BP could have been missed. Finally, the doses of eplerenone used by Ohtani et al. (14) (12.5 or 40 mg·kg^−1^d^−1^) and Nagata et al. (15) (30 or 100 mg·kg^−1^d^−1^) could be below those required for BP lowering and plasma exposure data for eplerenone were not provided in their studies. It is important to note that in our study, eplerenone was administered con-comitantly with salt-loading at adult age, and the salt content is lower than that employed in most of the reports (4,7,14,15).

With respect to mechanism of action, it is suggested that MR antagonists lower BP via enhanced natriuresis/diuresis or nonrenal mechanisms, including antagonizing MR in the central nervous system or cardiovascular tissues (34-37). In the present study, no natriuretic/diuretic effect of eplerenone was detected under either low-salt or high-salt conditions by weekly monitoring of renal excretory function. We suspect that an early effect on natriuresis/diuresis could have been missed by assessing renal excretory function after eplerenone had been administered for 1 week. Accordingly, we performed an acute study with eplerenone (100 mg·kg^−1^d^−1^ for 3 days) in age-matched Dahl SS rats (without telemetry device implantation), and determined a significant natriuretic effect on the first day of treatment in the low-salt diet condition but not in the high-salt diet condition. The initial natriuretic/diuretic effect of eplerenone in animals on a low-salt diet could lead to a leftward shift of the steady state pressure-natruresis relationship curve, whereby sodium balance is maintained at a lower BP level. It is notable that the BP lowering effect of eplerenone in animals on a high-salt diet is quite different from the effect seen in animals on a low-salt diet, and the anti-hypertensive effect of eplerenone in high-salt fed animals cannot be explained by acute changes in natriuresis/diuresis. However, the contributions of nonrenal mechanisms such as antagonizing MR in the brain, heart, and vasculature to its BP efficacy remain to be determined.

The second purpose of the present study was to evaluate the effects of eplerenone on organ damage. It is interesting that Dahl SS rats, even on a low-salt diet, developed proteinuria. This effect may be attributed to the glomerular phenotype (loss of foot processes in podocytes) of this rat strain (38). Although glomerular hypertension could accelerate proteinuria, the increase in glomerular pressure may not be a dominant contributor to the development of proteinuria in Dahl SS rats on low salt diet. Accordingly, a modest decrease in BP by eplerenone in Dahl SS rats on low salt diet, may not decrease urinary protein excretion. Animals on a high-salt diet developed more extensive renal injury as demonstrated by increased kidney injury biomarkers and histopathologic alterations in glomeruli, vasculature, and interstitium. The kidneys were enlarged, and proteinuria was dramatically accelerated. All the above-mentioned changes induced by a high-salt challenge were attenuated by eplerenone treatment. These findings further support the concept that eplerenone has renoprotective effects in salt-sensitive hypertensive renal injuries (7,8). With respect to cardiac structure and function, it is apparent that a high-salt diet caused cardiac hypertrophy and increased heart rate; the latter could potentially reflect an early stage of cardiac dysfunction. Chronic treatment with eplerenone significantly reduced the cardiac changes observed in animals on a high-salt diet. Consistent with our findings, Ohtani et al. (14) and Nagata et al. (15) observed that salt-loading could induce heart failure when 8% salt was introduced to 7 weeks old Dahl SS rats for 5-6 weeks, subsequent treatment with eplerenone for 7-8 weeks results in attenuation in left ventricular hypertrophy and heart failure. Thus, MR antagonism is beneficial to the heart under high-salt conditions. It is not the scope of the present study to investigate the mechanism of organ protection afforded by eplerenone. However, it is reasonable to speculate that hemodynamic and/or nonhemodynamic, as well as other direct actions, might contribute to beneficial effects of eplerenone on tissues. An extensive body of evidence suggests salt-sensitive hypertension is associated with endothelial dysfunction, oxidative stress, inflammation, and fibrosis (28-30), and eplerenone has been shown to suppress the induction of oxidative stress (4,7,15), fibrosis/apoptosis, and inflammation (7) in Dahl SS rats fed high-salt diet.

It is generally accepted that high-salt intake suppresses the renin-angiotensin system and aldosterone levels and low-salt intake has the opposite effect (39). Previous reports showed that salt-loading in Dahl SS rats reduced plasma aldosterone levels (8,14,15,40); however, tissue (heart and kidney) levels of aldosterone (40) or MR mRNA or protein levels of MR (8,14,15) were upregulated. These findings suggested that high dietary salt evokes inappropriate activation of the local aldosterone/MR pathway, which may play a causal role in the development of salt-sensitive hypertension. In the present study, high dietary salt did not change serum aldosterone levels, contrary to reports from other groups (8,14,15,34). The underlying reason for this divergent finding is unclear at this stage. However, we speculate that the blood sampling method and time of blood collection may affect the measurement of serum aldosterone. In the present study, blood samples were collected around 10AM by jugular venepuncture in conscious animals. It is known that aldosterone secretion exhibits circadian rhythm in rats (41,42) and humans (43,44). Preliminary data (unpublished) from our laboratory indicates that aldosterone levels measured in conscious animals are at their lowest in the early morning. In addition, the stress caused by the procedure of jugular venepuncture in a conscious state might have an impact on aldosterone secretion. However, it is difficult to make an appropriate comparison with other published studies in which blood collection was conducted under anesthesia and the timing of the sample collection was not reported (8,14,15,40). We did not determine tissue levels of aldosterone in the present study. Interestingly, Bayorh et al. (40) reported that tissue levels of aldosterone are increased under conditions of high-salt administration to Dahl SS rats. Thus, it is conceivable that the beneficial effects of eplerenone (hemodynamic or organ protection) may be a consequence of inhibiting the effects of elevated tissue levels of aldosterone.

A concern associated with the clinical use of MR antagonists is hyperkalemia (45). In the present study, urinary potassium excretion was slightly less in those animals that received eplerenone but the change did not reach statistical significance. Furthermore, plasma potassium levels were not statistically different among groups, although there was a trend for an increase in plasma potassium for those animals on a high-salt diet with eplerenone treatment. The somewhat modest effect on urinary potassium excretion and the absence of elevated plasma aldosterone in these studies may both be a consequence of submaximal MR blockade. Although we do not have a direct *in-vivo* measure of the extent of receptor blockade achieved, we do know that we achieved plasma levels of eplerenone of less than 100 nM (77.3 nM in animals on a low-salt diet and 90.6 nM in animals on a high-salt diet). Published data (37) indicated that in rats, the IC_50_ of eplerenone for the aldosterone receptor was 360 nM. Thus, plasma levels of eplerenone of less than 100 nM would not be expected to produce complete receptor blockade, which may account for the minimal changes in potassium excretion and little change in the plasma concentration of aldosterone. Furthermore, it is also possible that higher doses of eplerenone, and hence higher plasma exposure, could produce a greater extent of BP lowering and organ protection.

In conclusion, the findings of the present study demonstrate that MR antagonism attenuates the development and progression of hypertension and provides target organ protection in a model of salt-sensitive hypertension. Importantly, these data further support the promise of MR antagonists for the treatment of hypertension.
